# A CRISPR/Cas12a Based Universal Lateral Flow Biosensor for the Sensitive and Specific Detection of African Swine-Fever Viruses in Whole Blood

**DOI:** 10.3390/bios10120203

**Published:** 2020-12-10

**Authors:** Jinghua Wu, Omar Mukama, Wei Wu, Zhiyuan Li, Jean De Dieu Habimana, Yinghui Zhang, Rong Zeng, Chengrong Nie, Lingwen Zeng

**Affiliations:** 1 School of Food Science and Engineering, Foshan University, Foshan 528231, China; wu_jinghua@126.com (J.W.); zyinghui@163.com (Y.Z.); zrlp525@163.com (R.Z.); 2 Key Laboratory of Regenerative Biology, South China Institute for Stem Cell Biology and Regenerative Medicine, Guangzhou Institutes of Biomedicine and Health, Chinese Academy of Sciences, Guangzhou 510530, China; omar@gibh.ac.cn (O.M.); li_zhiyuan@gibh.ac.cn (Z.L.); hajado@yahoo.fr (J.D.D.H.); 3 Department of Applied Biology, College of Science and Technology, University of Rwanda, Avenue de l’armée, Kigali P.O. Box 3900, Rwanda; 4 University of Chinese Academy of Sciences, 19 Yuquan Road, Shijingshan District, Beijing 100049, China; 5 College of Food Science and Engineering, Qingdao Agricultural University, Qingdao 266109, China; wuweiouc@126.com; 6 Langyuan Biotechnology LLC, Foshan 528313, China

**Keywords:** African swine fever virus, target pre-amplification, CRISPR–Cas12a, lateral flow biosensor, detection

## Abstract

Cross-border pathogens such as the African swine fever virus (ASFV) still pose a socio-economic threat. Cheaper, faster, and accurate diagnostics are imperative for healthcare and food safety applications. Currently, the discovery of the Clustered Regularly Interspaced Short Palindromic Repeats (CRISPR) has paved the way for the diagnostics based on Cas13 and Cas12/14 that exhibit collateral cleavage of target and single-stranded DNA (ssDNA) reporter. The reporter is fluorescently labeled to report the presence of a target. These methods are powerful; however, fluorescence-based approaches require expensive apparatuses, complicate results readout, and exhibit high-fluorescence background. Here, we present a new CRISPR–Cas-based approach that combines polymerase chain reaction (PCR) amplification, Cas12a, and a probe-based lateral flow biosensor (LFB) for the simultaneous detection of seven types of ASFV. In the presence of ASFVs, the LFB responded to reporter trans-cleavage by naked eyes and achieved a sensitivity of 2.5 × 10^−15^ M within 2 h, and unambiguously identified ASFV from swine blood. This system uses less time for PCR pre-amplification and requires cheaper devices; thus, it can be applied to virus monitoring and food samples detection.

## 1. Introduction

African swine fever (ASF) is a highly contagious, fatal infectious disease of domestic and wild pigs caused by an Asfarviridae family DNA virus called the African swine fever virus (ASFV) [1]. It can be expressed in three main forms: acute, subacute, and chronic [2,3]. ASFV has a morbidity and mortality rate of up to 100% and is widely prevalent in 13 different countries and regions around the world since its discovery in Kenya in 1921 [4,5]. ASFV is still a major problem in countries worldwide, including developing and developed countries such as Russia and countries in Western Europe [6,7,8]. According to the China Center for Animal Health and Epidemiology, ASFV epidemics have currently caused severe loss to China’s livestock industry [9]. 

At present, the detection of ASFV can be divided into two categories according to different diagnostic tests: one is detecting antibodies; the other is the detection of viral DNA. Conventional detection methods are serological enzyme-linked immunosorbent assay (ELISA) [10] and DNA-based, like polymerase chain reaction (PCR) [11]. These methods are robust, but they are still hindered by cost, reproducibility, and convenience and are prone to false-positive results [12,13]. These disadvantages can hinder their in-depth application in on-site testing. Therefore, it is imperative to develop rapid, accurate, and non-cross-contamination detection technologies.

Recently, CRISPR–Cas12/Cas13/Cas14 systems have been exploited in the analytical method field and have been found suitable for detecting various targets, including bacteria, drug resistance genes, human DNA genotypes, and cancer mutations [14,15,16,17,18]. They possess a unique trans-cleavage property of target nucleic acids and non-specific single-stranded DNA (ssDNA) (referred to hereafter as a reporter) in the vicinity [16,18,19,20]. For example, DETECTR [18] and HOLMES [15,16] detection systems used crRNA-guided Cas12a enzymes for ultrasensitive and rapid detection of target DNA, and SHERLOCK [17] for target RNA. Clustered Regularly Interspaced Short Palindromic Repeats (CRISPR) systems combined with pre-amplification methods such as PCR [15] and recombinase polymerase amplification (RPA) [17,18] increase sensitivity and reduce the possibility of false results. The high specificity of CRISPR-–Cas12a relies on the fact that it doesn’t initiate cleavage of the reporter nucleic acid if there is a mismatch(es) between the target DNA sequence and the gRNA. The false-positive usually derives from cross-contamination. CRISPR/Cas12a exhibits collateral cleavage of target DNA and reporter, meaning that it can reduce cross-contamination in the laboratory since the amplified target is cleaved by Cas12a in the reaction tube. Though this method is expected to bring about a revolutionary breakthrough in molecular diagnostics, all the existing techniques use expensive fluorescence-based instruments and antibody-based lateral flows, which are still costly and prone to false-positive results. Thus, we aimed to develop a cost-effective gold nanoparticle-based lateral flow biosensor [21] with reduced background, contamination, bench-work, and expertise.

**Figure 1:**
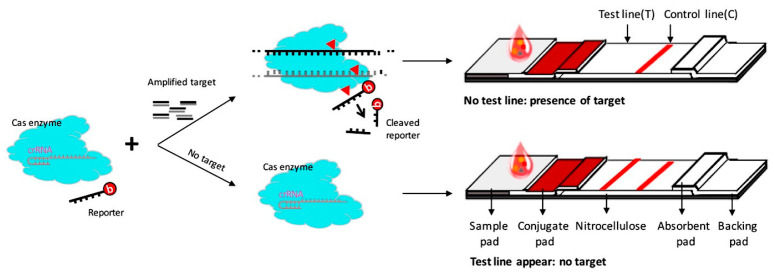
Illustrative principle of CRISPR/Cas–LFB assay. Target DNA from pre-extracted real samples is polymerase chain reaction (PCR) pre-amplified. The amplicons are applied to Cas12a reaction that contains specific crRNA, activator, and biotinylated single-stranded DNA (ssDNA) reporter. In the presence of the target, Cas12a trans-cleaves the biotinylated ssDNA reporter, which results in short sequences that cannot bind a DNA probe pre-immobilized on the lateral flow biosensor (LFB) test line, resulting in an undetectable test line on the LFB. The control line appears due to the excess of gold nanoparticles-streptavidin (AuNPs-SA) conjugate complex captured by the biotin-labeled goat anti-mouse IgG immobilized on the control line.

Here, we present a CRISPR/Cas–LFB assay, an ultrasensitive and specific lateral flow biosensor (LFB) that can respond to cleaved ssDNA reporter from crRNA-guided Cas12a cleavage of ASFV DNA target. The principle is that ASFV-containing samples were pre-amplified by PCR to enhance sensitivity. The amplified product was then added to the Cas12a detection system (including crRNA, Cas12a protein, and biotinylated ssDNA reporter). The Cas12a and crRNA complex recognized and cleaved the target DNA and subsequently triggered the biotinylated ssDNA reporter trans-cleavage, which cannot hybridize to its complementary DNA immobilized at the LFB test line (Figure 1). The CRISPR/Cas–LFB assay achieved excellent sensitivity and could identify ASFV in blood samples. Thus, we succeeded in unveiling a new CRISPR-based lateral flow biosensor for rapid and cost-effective identification of infectious diseases.

## 2. Materials and Methods

### 2.1. Materials, Chemicals, Reagents, and Methodology

All oligonucleotides, including the ASFV specific target sequence, primers, crRNAs (designed using benchling), reporters, and lateral flow capture probes were synthesized by Sangon Biotech (Shanghai, China) (Table 1). Biotin-labeled goat anti-mouse IgG (H+L) dispensed on the control line was from Beyotime Biotechnology (Shanghai, China). LbaCas12a, NEB buffer 3.1, Bst 2.0 WarmStart polymerase was purchased from New England Biolabs (Ipswich, MA, USA). Deoxynucleoside triphosphates (dNTPs) and Taq polymerase premix were purchased from Takara (Beijing, China).

Gold trichloride acid (HAuCl_4_.3H_2_O), trisodium citrate, and streptavidin (SA) were purchased from Sigma–Aldrich (Steinheim, Germany). Absorbent and fiberglass papers and nitrocellulose membranes were purchased from Sartorius AG (Gottingen, Germany). A dispenser to immobilize the biotin-labeled goat anti-mouse IgG and probe DNA on the LFB and a nitrocellulose membrane cutter were purchased from Shanghai Kinbio (Shanghai, China). A portable strip reader was from Goldbio Technology Co. (Shanghai, China). Genome and plasmid extraction kits were purchased from Qiagen (Hilden, Germany) and Tiangen Biotech (Beijing, China), respectively. All buffers used in this study were prepared in our laboratory. Other chemicals were purchased from standard commercial sources and were of pure analytical grade.

### 2.2. Target DNA Design, Virus Collection, and DNA Extraction

In this study, two types of ASFV target DNA were used: (1) a 270 bp ASFV specific sequence targeting conserved regions of VP73 genes of 7 different types of ASFV [11] synthesized and cloned in pUC57 vector used for study design and positive control; and (2) ASFV-positive and ASFV-negative blood samples pre-identified using PCR. The pUC5-ASFV clone and DNA from whole blood samples were extracted using Tiangen Biotech (Beijing, China) and Qiagen Blood Kit (Beijing, China), respectively. The extracted DNAs were used for CRISPR/Cas-LFB assay.

### 2.3. PCR Amplification

For PCR amplification, 25 µL PCR premix was composed of 1 µL forward and reverse primers (0.3 µM), 12.5 µL 1 × rTaq Premix, 2 µL of extracted genomic DNA (200 ng), and ddH_2_O to final reaction volume. The reaction mixture was incubated in a thermocycler using 3 steps PCR protocol for 45 min: 95 °C, 5 min for denaturation; 95 °C, 10 s for each cycle; 55 °C, 10 s for annealing; 72 °C, 30 s for elongation; 35 cycles. PCR products were analyzed using 2.5% agarose gel electrophoresis.

### 2.4. Target Cleavage Assay

For CRISPR/Cas-LFB assay analysis of trans-cleaved reporters, the cleavage assay was carried out according to [18] with slight modifications. The LbaCas12a complex was preassembled as follows: 12 µL of cleavage buffer (150 mM KCl, 10 mM MgCl_2_, 1% glycerol, 0.5 mM DTT, 20 nM HEPES (pH 7.5)) or 3 µL NEB buffer 3.1, 3 µL crRNA (300 nM), 1 µL LbaCas12a (36 nM), and 5 µL activator (40 nM) incubated at 37 °C for 10 min. Then, an LbaCas12a detection was prepared using 21 µL LbaCas12a-crRNA-activator complex, 2 µL biotinylated ssDNA reporter (20 nM), and 2 µL extracted target DNAs. Finally, the reaction was incubated at 37 °C for 30 min and analyzed using LFB after adding ddH_2_O up to 60 µL. The reporter trans-cleavage was monitored by the naked eye on the LFB within 10 min, and an LFB reader provided peak area values of the test and control lines’ signal intensities. The test lines’ peak area intensities were plotted after LFB photo-analysis using Image J (NIH Research Services, Bethesda, MD, USA), which provided similar intensities between each analyzed triplicate.

For fluorescence analysis of trans-cleavage reaction, 50 µL reaction set-up was prepared as mentioned above with 5′FAM and 3′TAMRA labeled ssDNA reporter instead of biotinylated ssDNA reporter. The reaction was incubated and proceeded at 37 °C in a 96-wells microplate reader Mithras^2^ LB 943 (Berthold Tech, Bad Wildbad, Germany). The fluorescence from reporter sequence cleavage was measured every 5 min from 0 to 1 h with detection at λ excitation = 495 and λ emission = 520.

### 2.5. Construction of the LFB

The LFB was composed of 3 main parts: (1) a sample pad (16 mm in width) prepared using immersion buffer (1% Triton, 1% BSA, 2% glucose, 50 mM boric acid, pH 8.0), dried and stored in a desiccant container. (2) a conjugate pad containing AuNPs prepared according to the previous method [22,23,24], which were conjugated to SA (0.5 mg/mL) using 1 mL of AuNPs solution concentrated four times by centrifugation (12,000 rpm, 25 min) in 100 µL of suspension buffer (20 mM Na_3_PO_4_, 5% BSA, 0.25% Tween-20, 10% sucrose, and 0.1% NaN_3_). The solution was gently shaken for 3 h at 4 °C, centrifuged (12,000 rpm, 25 min), and rinsed three times with suspension buffer to remove unbound SA. The red pellet was resuspended in 100 µL of suspension buffer before being dispensed on the glass fiber conjugate pad. (3) The nitrocellulose membrane consisted of the capture probe (complementary to ssDNA reporters) and biotin-labeled goat anti-mouse IgG dispensed on the LFB nitrocellulose membrane strip (25 mm in width) to form test and control zones, respectively. The LFB parts were dried at room temperature for 12 h and stored in a desiccant container. All pads were sequentially assembled along the adhesive part of the nitrocellulose membrane with an overlap of 2 mm and then cut into 0.4 cm-wide strips.

### 2.6. ASFV Clinical Samples Preparation and Analysis

To confirm our approach’s applicability, we used ASFV-negative and ASFV-positive whole blood samples collected and preserved at 4 °C in EDTA tubes in Prof. Liangxue Lai’s swine blood collection in Guangzhou Institutes of Biomedicine and Health. ASFV genomic DNA was extracted using Qiagen Blood Kit without pretreatment from 200 µL of each clinical sample. Two microliters of each extracted DNA were subjected to PCR and then applied to Cas12a cleavage assay.

### 2.7. Statistical Analysis

Each result presented is the mean ± standard error of the mean (SEM) of the LFB test line’s peak area readings from the triplicates.

## 3. Results

### 3.1. Overview of the CRISPR/Cas–LFB Detection System

**Figure 2:**
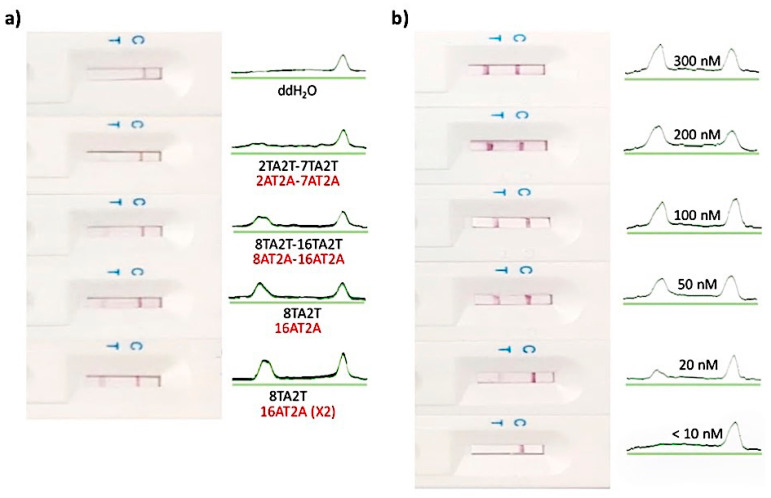
Optimization of reporter length and concentration. (**a**) Different length of 100 nM water-dissolved reporter sequences (in black) applied on the LFB test line complementary probe (in red). (**b**) Different concentrations of 8TA2T reporter applied on the LFB with 16AT2A (×2) complementary probe.

As illustrated in Figure 1, the principle of the CRISPR/Cas–LFB approach is based on PCR pre-amplification to improve sensitivity through target DNA pre-amplification and the detection of trans-cleaved biotinylated ssDNA reporter upon target recognition by Cas12a effector. As previously reported [25], from a wide range of screened reporter sequences length, the 5′biotin-TTTTTTTTATT-3′ reporter (11 bases) successfully hybridized with its complementary test line where the capture probe (38 bases) immobilized on the LFB test line, allowing it to be detectable by the naked eye (Table 1, Figure 2a). The test line was not observed when ddH_2_O and shorter reporter sequences were applied (Figure 2a). The test line intensity also depended on the reporter’s concentration (10, 20, 50, 100, 200, 300 nM), of which 50 nM was chosen for all downstream experiments, since it was the concentration that provided a strong test line in the absence of the target, and a non-detectable test line signal in the presence of the target (Figure 2a). In the presence of the target, trans-cleaved reporters were not detected on the test line due to the reporter length reduction that cannot bind the test line. The biotin-labeled goat anti-mouse IgG immobilized on the control line captured the excess AuNPs-SA conjugate complex, displaying a red band that indicated that the LFB works perfectly.

### 3.2. The Feasibility and Sensitivity Evaluation of CRISPR/Cas–LFB Assay Using Recombinant Plasmid

**Figure 3:**
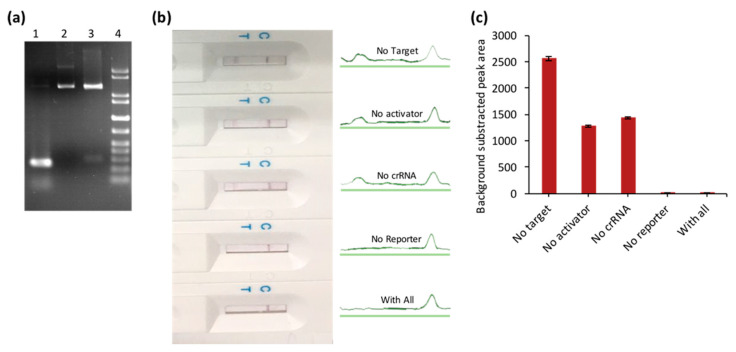
The feasibility assay of CRISPR/Cas–LFB assay using a recombinant plasmid. (**a**) Gel electrophoresis of the PCR-amplified 270 bp of African swine fever virus (ASFV)-specific sequence (1) cloning in pUC57 (2) and double digestion verification at *BamHI*/*EcoRI* enzyme site (3). (**b**) CRISPR/Cas–LFB reaction in the presence/absence of different reaction components showing test and control line signal intensities. (**c**) Bar chart of the LFB test lines in (**b**).

The system was first studied using pUC57-ASFV recombinant plasmid DNA target (Figure 3a). We selected the conserved region of ASFV target genes (VP73) (Table 1) to target 7 different ASFV types simultaneously [11]. PCR pre-amplification of ASFV target was performed for 45 min (Figure 3a) and applied to Cas12a reaction-specific components (crRNA, activators, and biotinylated ssDNA reporter). The CRISPR/Cas12a–LFB assay identified the target plasmid’s presence accurately and efficiently through an undetectable LFB test line. In contrast, in the absence of the critical reaction components, a visible LFB test line was observed (Figure 3b,c).

**Figure 4:**
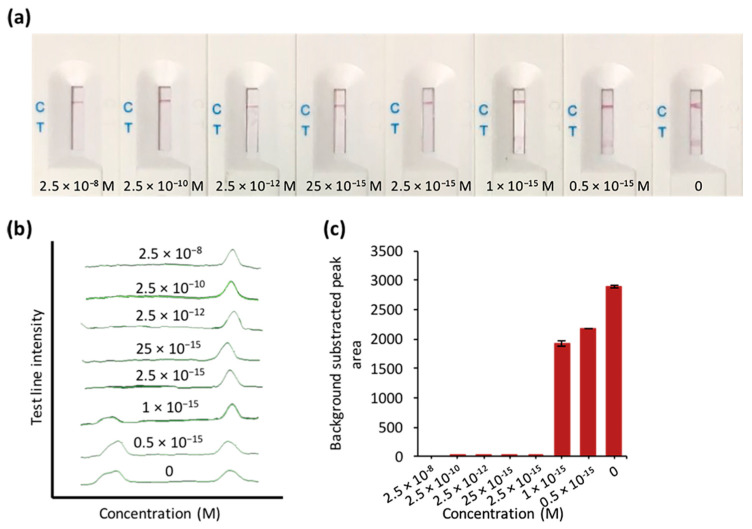
Evaluation of sensitivity using different concentrations of the recombinant plasmid. (**a**) LFB responses for the different concentrations. (**b**) Test and control line signal intensities. (**c**) Bar chart of the LFB test lines in (**a**,**b**). 0 stands for negative control.

To evaluate the sensitivity, different concentrations of the serially diluted recombinant plasmid (2.5 × 10^−8^–0.5 × 10^−15^ M) were tested using both PCR and CRISPR/Cas–LFB. PCR alone achieved >25 × 10^−15^ M sensitivities (Figure S1). However, CRISPR/Cas–LFB combined with PCR pre-amplification showed no detectable test line as a result of the reporter trans-cleavage (Figure 4). As low as 2.5 × 10^−15^ M (~1554.46 copies/µL) was detected. Our biosensor’s qualitative activity was also confirmed by the previously reported fluorescence-based CRISPR diagnostics assays [15,16] using different concentrations of recombinant plasmids, which were more sensitive compared to our assay (Figure S2). However, these detection methods require fluorescence apparatuses and expensive antibody-based strips [24,25]. Moreover, our CRISPR/Cas–LFB can be combined with isothermal amplification to reduce cost and facilitate on-site deployment.

### 3.3. Selectivity of the Biosensor for ASFV Detection

**Figure 5:**
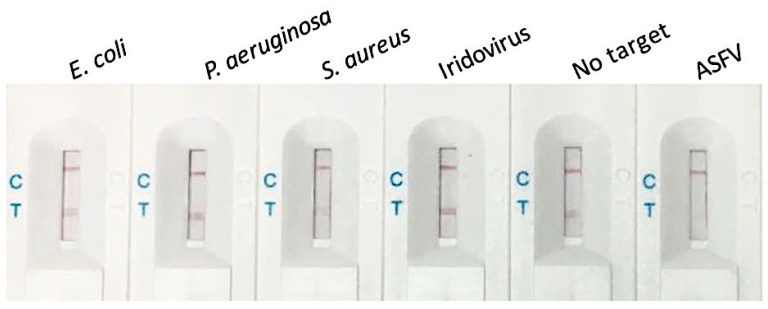
Selectivity assay using different strains. Biosensor images with test and control lines responses corresponding to the subjected 10^4^ cfu/mL strains.

The selectivity assay consisted of various bacteria strains (*Escherichia coli, Pseudomonas aeruginosa*, *Staphylococcus aureus*), viruses (Iridovirus, ASFV), and co-incubation in the presence of ASFV. Except for the absence of ASFV, which showed similar detectable test lines, the distinctive cleavage was only observed in the presence of ASFV (Figure 5). The co-incubation of the pre-mentioned strains with ASFV showed no test lines due to the reporter trans-cleavage (Figure S3), confirming our method’s high specificity.

### 3.4. The Application of CRISPR/Cas–LFB Assay in Real Samples

**Figure 6:**
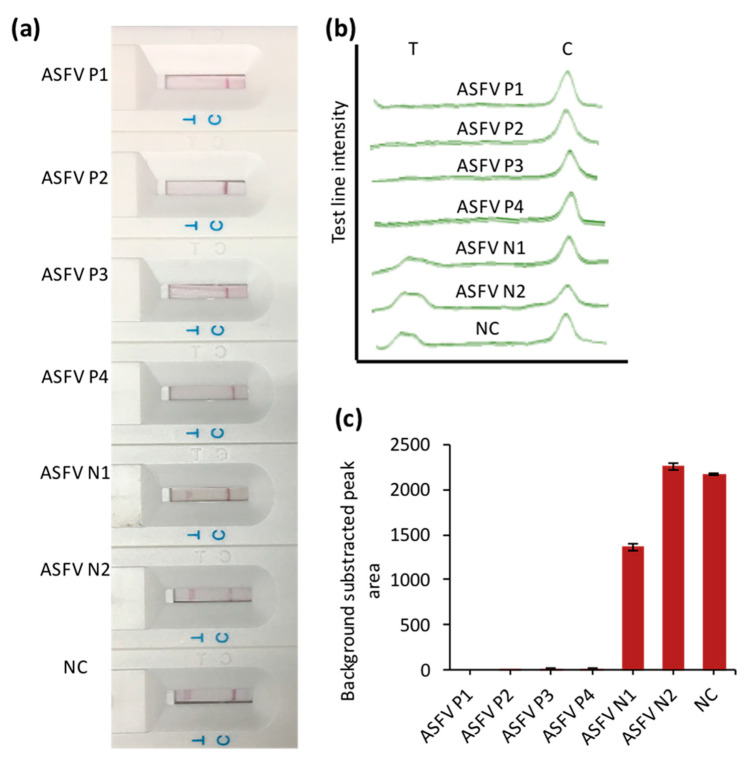
Clinical samples testing. (**a**) LFB responses for the positive (ASFV P1–P4) and negative (ASFV N1–N2) clinical samples. (**b**) Test and control line signal intensities. (**c**) Bar chart of the LFB test lines in (**a**,**b**). NC stands for negative control (water).

A total of 6 available swine blood samples with 4 ASFV-positive and 2 ASFV-negative were confirmed by PCR (Figure S4) and examined blindly using CRISPR/Cas–LFB assay (Figure 5). As shown in Figure 6, no detectable test line was observed from the positive samples. In contrast, a clear test line was detected in negative samples (Figure 6a), indicating our biosensor’s high specificity. These results were confirmed by detectable peak intensities and areas (Figure 6b). This activity was also confirmed using PCR amplification, which corroborated our assay (Figure S4).

## 4. Discussion

ASFV is one of the most detrimental viruses occurring in swine, with high morbidity and mortality, causing severe socio-economic concerns. The early detection of ASFV is crucial for early control, monitoring, and treatment. Traditional detection methods such as ELISA and PCR-based methods have been utilized, but they are still laborious, costly, and non-deployable. Nucleic acids isothermal amplification methods are currently considered promising in the rapid detection of ASFV. Nevertheless, carry-over cross-contamination, tedious gel electrophoresis analysis, and toxic dyes still pose difficulties. The currently developed CRISPR–Cas-based detection systems promise potential diagnosis of broad-range nucleic acids [15,16,17,18,19,20]. However, all these approaches employ expensive fluorescence-based apparatuses or monoclonal antibody-based lateral flow biosensors. Our developed lateral flow biosensor approach is readily available, inexpensive, and universal for detecting ASFV and other infectious diseases. By targeting the conserved region of VP73 genes in seven different ASFV strains with PCR amplification-specific primers and Cas12a-based biosensors, we unambiguously identified ASFV from swine blood samples with no false results or expensive apparatuses, indicating the applicability of our technique. Our CRISPR/Cas–LFB method can detect around 1000 copies per µL within 2 h, comparable to the existing traditional methods and CRISPR–Cas diagnostics [15,17,18].

Most importantly, our designed probe-based biosensor is universal for any singleplex CRISPR–Cas detection. Although PCR is a mature and commonly used method, its on-site availability in resource-limited areas is limited; thus, isothermal amplification methods such as loop-mediated isothermal amplification and recombinase polymerase could be integrated with our CRISPR/Cas–LFB. In addition, a multiplexing biosensor can also be designed to detect other pathogens simultaneously by using their specific reaction components.

## Figures and Tables

**Table 1:** Nucleic acids used in this study.

Name	Sequence (5′-3′)
**Target ASFV DNA**	TGTGAACAAAAGTTATGGGAAACCCGACCCCGAACCCACTTTGA GTCAAATCGAAGAAACACATTTGGTTCATTTTA**ATGCGCATTTT** **AAGCCTTATGTTC**CAGTAGGGTTTGAATACAATAAAGTACGCCC GCATACGGGTACCCCCACCTTGGGAAACAAGCTTACCTTTGGTAT TCCCCAGTACGGAGACTTTTTCCATGATATGGTGGGCCACCATAT ATTGGGTGCATGTCATTCGTCCTGGCAGGATGCTCCGATTCAGGG CAC
**PCR primers** [11]	
**Forward**	AGTTATGGGAAACCCGACCC
**Reverse**	CCCTGAATCGGAGCATCCT
**crRNA**	UAAUUUCUACUAAGUGUAGAU**ATGCGCATTTTAAGCCTTATG** **TTC**
**Activator (24 bp)**	TTTGGTTCATTTTAATGCGCATTTT
**Reporter**	Biotin-TTTTTTTTATT
**Test-line probe (38 bases)**	AATAAAAAAAAAAAAAAAAAATAAAAAAAAAAAAAAAA

Underline represents palindromic adjacent motif (PAM) region. Bold represents crRNA binding sequence

## Supplementary Materials

The following are available online at https://www.mdpi.com/2079-6374/10/12/203/s1, Figure S1: Sensitivity assay using different concentrations of recombinant plasmids (ng/µL), Figure S2: Fluorescence-based assay using different concentrations of recombinant plasmids, Figure S3: Selectivity assay using different strains co-incubated with ASFV, Figure S4: PCR amplification confirmation of 6 ASFV clinical samples.
