# Supplementary material for: A CRISPR/Cas12a Based Universal Lateral Flow Biosensor for the Sensitive and Specific Detection of African Swine-Fever Viruses in Whole Blood
Source: Biosensors (Basel). 2020 Dec 10;10(12):203. doi: 10.3390/bios10120203 (PMC7763806; doi:10.3390/bios10120203)
Supplement: Supplementary file 1 [file biosensors-10-00203-s001.pdf]

A CRISPR/Cas12a Based Universal Lateral Flow Biosensor for the Sensitive and Specific Detection of African Swine-Fever Viruses in Whole Blood

Jinghua Wu [1,†], Omar Mukama [2,3,4,†], Wei Wu [5], Zhiyuan Li [2], Jean De Dieu Habimana [2], Yinghui Zhang [1], Rong Zeng [1], Chengrong Nie [1,*] and Lingwen Zeng [1,6,*]

1 School of Food Science and Engineering, Foshan University, Foshan 528231, China; wu_jinghua@126.com (J.W.); zyinghui@163.com (Y.Z.); zrlp525@163.com (R.Z.)
2 Key Laboratory of Regenerative Biology, South China Institute for Stem Cell Biology and Regenerative Medicine, Guangzhou Institutes of Biomedicine and Health, Chinese Academy of Sciences, Guangzhou 510530, China; omar@gibh.ac.cn (O.M.); li_zhiyuan@gibh.ac.cn (Z.L.); hajado@yahoo.fr (J.D.D.H.)
3 Department of Applied Biology, College of Science and Technology, University of Rwanda, Avenue de l'armée, Kigali P.O. Box 3900, Rwanda
4 University of Chinese Academy of Sciences, 19 Yuquan Road, Shijingshan District, Beijing 100049, China
5 College of Food Science and Engineering, Qingdao Agricultural University, Qingdao, 266109, China; wuweiouc@126.com
6 Langyuan Biotechnology LLC, Foshan 528313, China
* Correspondence: niecr@126.com (C.N.); zeng6@yahoo.com (L.Z.)
† These authors contributed equally.

Supplementary Materials:

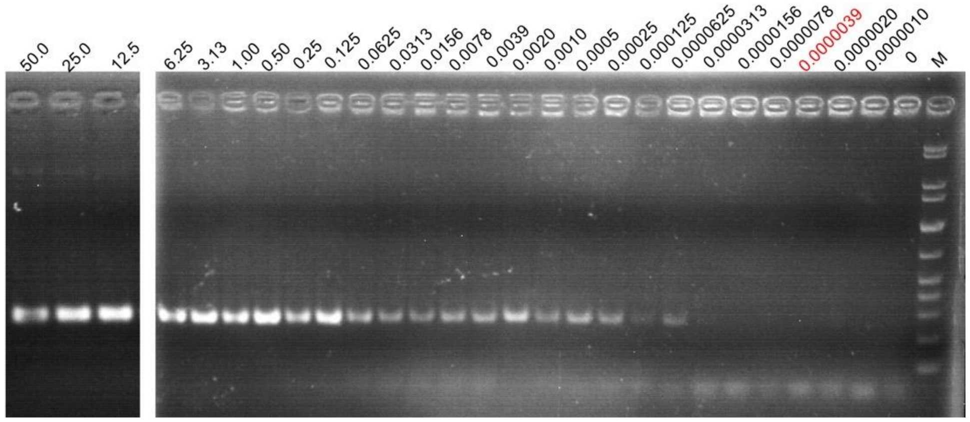

Figure S1. Sensitivity assay using different concentrations of recombinant plasmids (ng/μl). M stands for 1kb marker, while red is the sensitivity achieved by CRISPR/Cas-LFB method.

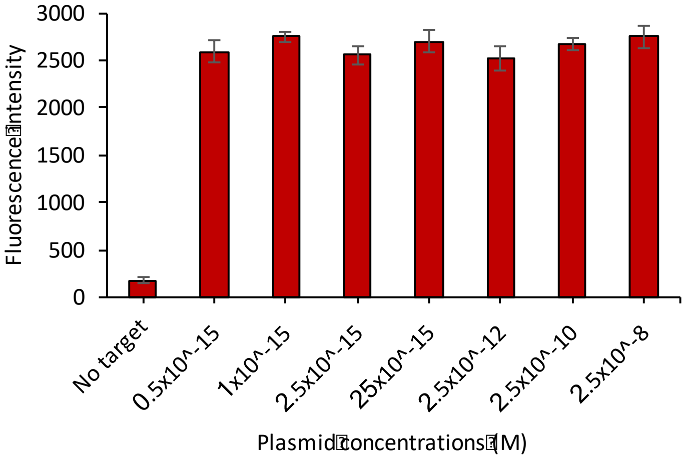

Figure S2. Fluorescence-based assay using different concentrations of recombinant plasmids.

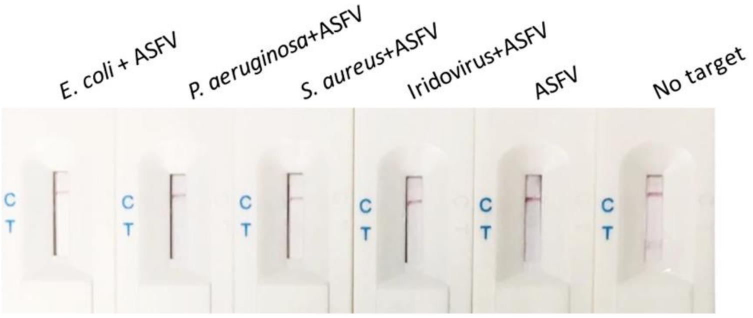

Figure S3. Selectivity assay using different strains co-incubated with ASFV. Biosensor images with test and control lines responses corresponding to the subjected 10^4 cfu/ml strains.

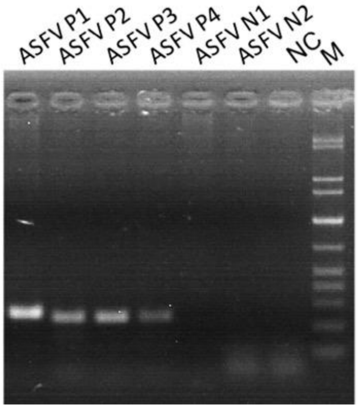

Figure S4. PCR amplification confirmation of 6 ASFV clinical samples. ASFV P1-P4: ASFV positives; ASFV P1-P2: ASFV negatives; NC: negative control; M: 1kb marker.
